# TNF-α and INF-γ primed canine stem cell-derived extracellular vesicles alleviate experimental murine colitis

**DOI:** 10.1038/s41598-020-58909-4

**Published:** 2020-02-07

**Authors:** Ju-Hyun An, Qiang Li, Dong-Ha Bhang, Woo-Jin Song, Hwa-Young Youn

**Affiliations:** 1 Labolatory of Veterinary Internal Medicine, Department of Veterinary Clinical Science, College of Veterinary Medicine and Research institute for Veterinary Science, Seoul National University, 1 Gwanak-ro, Gwanak-gu Seoul, 08826 Republic of Korea; 2 Department of Molecular Cell Biology, Samsung Biomedical Research Institute, Sungkyunkwan University School of Medicine, Suwon-si, Gyeonggi-do 16419 Republic of Korea

**Keywords:** Mesenchymal stem cells, Mesenchymal stem cells, Stem-cell therapies, Stem-cell therapies

## Abstract

The inflammatory bowel diseases (IBD) are characterized by relapsing inflammation and immune activation diseases of the gastrointestinal tract. Extracellular vesicles, which elicit similar biological activity to the stem cell themselves, have been used experimentally to treat dextran sulfate sodium (DSS)-induced colitis in murine models though immunosuppressive potential. In this study, we investigated whether the Extracellular vesicles (EVs) obtained by stimulating inflammatory cytokine on canine adipose mesenchymal stem cells (cASC) improved anti-inflammatory and/or immunosuppressive potential of EVs, and/or their ability to alleviate inflammation in colitis. We also explored the correlation between immune cells and the inflammatory repressive effect of primed EVs. Pro-inflammatory cytokines such as TNF-α and IFN-γ increased immunosuppressive protein such as HGF, TSG-6, PGE2 and TGF-β in EVs. Moreover, the anti-inflammatory effect of EVs was improved through pretreatment with inflammatory cytokines. Importantly, EVs obtained from primed stem cells effectively induced macrophage polarization toward an anti-inflammatory M2 phenotype and suppressed activated immunity by enhancing regulatory T cells in inflamed colon in mice. Our results provide a new and effective therapy for the EVs obtained from ASC stimulated with TNF-α and IFN-γ against not only IBD, but also immune-mediated disease.

## Introduction

Inflammatory bowel disease (IBD) is a comprehensive term used to describe diseases related to the chronic inflammation of the gastrointestinal tract^1^. The exact cause of IBD is unknown, but the immune system responds incorrectly to environmental triggers, which causes inflammation of the gastrointestinal tract. In addition to lowering the quality of life, IBD is likely to progress to rectal cancer if not managed properly, which increases the risk of death in IBD patients and results in a mortality rate of 15% among IBD patients^2^. Despite recent advances in antibiotic therapies and immune suppressors^3,4^, there is an unmet clinical need for IBD treatment strategies.

One promising strategy for the management of IBD is the use of extracellular vesicles from adipose tissue derived mesenchymal stem cell (ASC-EVs)^5^. EVs are a kind of membrane lipid vesicles with 30–100 nm in diameter, and they were previously thought to be metabolic produced of cells^5^. The functions of ASC-EVs are a subject of ongoing investigation. Although the therapeutic mechanisms underlying stem cell released EVs have not yet been elucidated, which elicit similar biological activity to the stem cells themselves^6^. Being taken up^7^, EVs are known to promote the activation of immune regulatory pathways by transferring proteins, mRNAs, and microRNAs (miRNAs) to target cells, which induce phenotypic and functional changes^8^.

In previous studies, exposure of stem cells to inflammatory cytokines increased the immune suppressive activity by stimulating the production of inhibitors of inflammations, such as prostaglandin E_2_(PGE_2_)^9^, TGF- β, HGF^10^ and TSG-6^11^. In addition, we found that conditioned medium (CM) of cultured canine stem cells primed with tumor necrosis factor-alpha (TNF-α) and interferon-gamma (IFN-γ) increase ability to relieve inflammation by pro- and anti-inflammatory cytokine modulation in peripheral blood mononuclear cells and macrophage cell lines^12^. Recently, it has been proposed that the predominant way by which stem cells participate is through a paracrine activity^13^, and it has been suggested that extracellular vesicle plays a major role in this paracrine activity^6^ thus playing a role in immunoregulation.

These evidences suggested that by adjusting the culture conditions of stem cells, it is possible to enhance or inhibit certain functions of EVs secreted from stem cells^14–16^. This has led to increased interest in EVs generated from stem cells exposed to inflammatory conditions^17^. Moreover, several research groups have shown that EVs generated from inflammatory cytokine-stimulated stem cells provide beneficial effects against a variety of *in vitro* and *in vivo* pathological conditions^18,19^. Domenis *et al*. showed that an inflammatory stimulus activate stem cells and can induce the released of EVs immunosuppressive abilities, since they are able to polarized macrophage towards the anti-inflammatory M2 phenotype^17^. Cosenza *et al*. showed that IFN-γ primed stem cells derived EVs were more efficient in suppressing inflammation in inflammatory arthritis model^16^. While much interest in ASCs-EVs for the treatment of many diseases has been shown, little is known on their exact function. Moreover, no study has evaluated the role of primed ASCs-derived EVs in pre-clinical models relevant for DSS-induced colitis.

The present study investigated the immunomodulatory properties of EVs released by canine ASCs after stimulation with TNF-α and IFN-γ to evaluate the influence of the inflammatory environment on the function of ASC-derived EVs. We also assessed the effect of EVs on the anti-inflammatory activity of M2 macrophages and regulatory T cells (Tregs) in an animal model of dextran sulfate sodium (DSS)-induced colitis. These findings suggest that canine ASCs pretreated with TNF-α and IFN-γ secreted EVs with enhanced immunoregulatory activities and that these EVs may be an effective therapeutic agent for the management of colitis as well as immune-mediated inflammatory diseases.

## Results

### Primed EVs improved the immune regulation of inflamed colons

**Figure 1:**
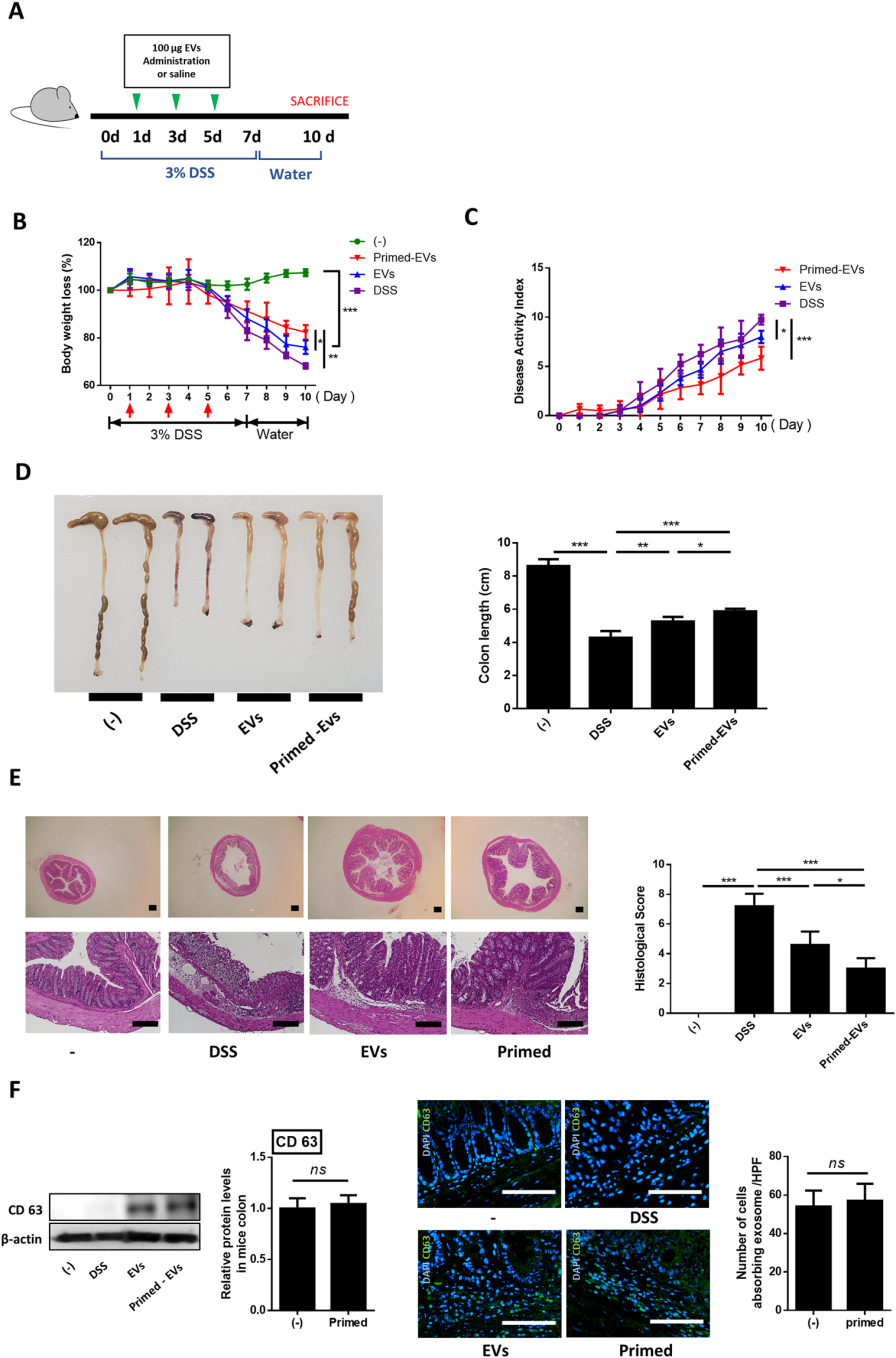
cASC-derived EVs ameliorates DSS-induced colitis in mice. Naïve or primed EVs (100 μg in 100 μl PBS) or vehicle control (100 μl PBS) were intraperitoneally (IP) injected into intraperitoneally on days 1, 3 and 5 after mice fed with 3% DSS. (**A**) Experimental scheme of DSS administration. (**B**) Mice were monitored for body weight. Value are calculated as percent of body weight from day 0. (**C**) The disease activity index (DAI) were monitored described for 10 days. (**D**) Colon length were assessed at day 10. (**E**) H&E staining of the colon section and histological score are shown. The primed EVs effectively inhibits inflammatory response in the inflamed colon. (**F**) Canine EVs levels in colon were detected by using western blot analysis. canine EVse was not found in naive group and PBS group, while EVs was confirmed in group injected with EVs. Immunofluorescent staining showed that a number of cells with EVs in the cytoplasm were identified in the mucosa and submucosa of colon. Black Bars = 100 μm. White Bars = 200 μm. Results were shown as mean ± standard deviation (*P < 0.05, **P < 0.01, ***P < 0.001, ****P < 0.0001 by one-way ANOVA analysis).

Using an established model, we investigated the effects of primed EVs on DSS-induced colitis. Accordingly, IBD mice began showing signs of diarrhea and rectal bleeding within four days of DSS exposure, and they began to lose weight and show signs of depression within five days. However, treatment with EVs significantly inhibited weight loss, and the severity of IBD-related clinical symptoms, such as fecal consistency and bloody diarrhea (Fig. 1B,C). The length of the colon was also longer in EVs-treated than in untreated IBD mice, and this effect was enhanced by primed EVs (Fig. 1D). Histologically, colon tissue of IBD mice showed signs of a loss of epithelial integrity accompanied by increased inflammatory cell infiltration. In contrast, EVs treatment rescued the integrity of colonic tissues and reduced inflammatory cell infiltration. Administration of primed EVs was associated with a further improvement (Fig. 1E). In addition, as a result of checking the content of EVs in colon through western blot, canine EVs was not found in naive group and PBS group, while EVs was confirmed in group injected with EVs. Moreover, in the mucosa and submucosa, in the group injected with EVs, a number of cells with EVs in the cytoplasm were identified (Fig. 1F).

### Effect of TNF-α/IFN-γ stimulation on cASCs and characterization of cASC

We examined whether inflammatory cytokine stimulation affects cASC morphology or viability after stimulation of cASC with TNF-α and IFN-γ for 24 h. No significant differences in morphology or viability were observed between the naive and primed cASCs (Supplementary Fig. S1A,B). Flow cytometric analysis demonstrated that very few of both stem cells expressed the known hematopoietic markers, CD34 and CD45, but that >95% expressed the known stem-cell markers, CD29, CD44, CD73, and CD90 (Supplementary Fig. S1C). Furthermore, both stem cells were found to be capable of differentiating into adipocytes, osteocytes, and chondrocytes (Supplementary Fig. S1D). These results confirmed that stem cell differentiation and surface markers are maintained following stimulation with inflammatory cytokines. However, we observed significant increases in the expression of TSG-6, TGF-β, HGF, and COX-2 genes in primed compared to naïve cASCs (Supplementary Fig. S1E).

### The effect of TNF-α and IFN-γ on the immunomodulation of cASCs and EVs

**Figure 2:**
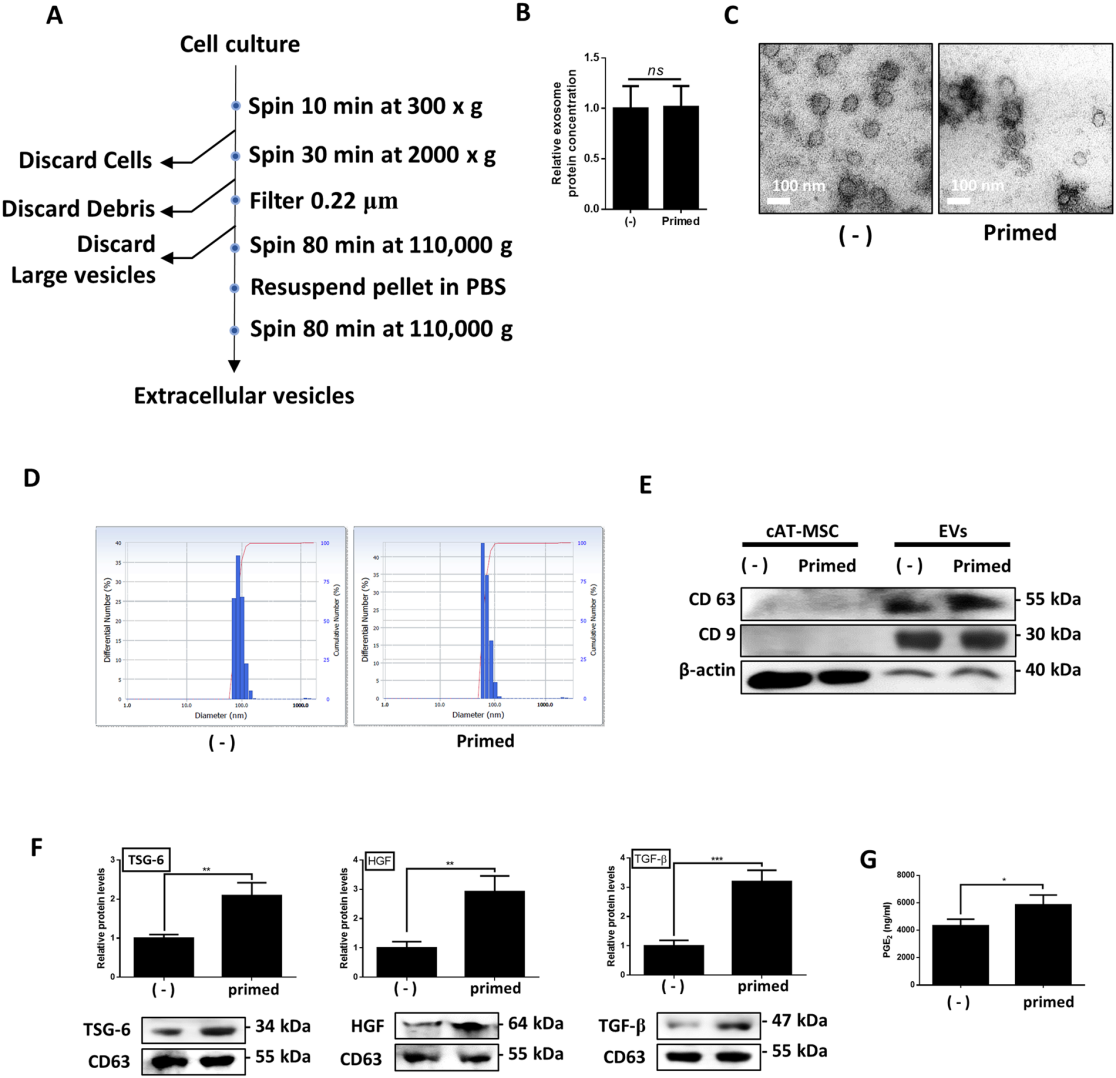
Characterization and immunomodulatory factors naïve- and primed cASCs derived EVs. (**A**) Scheme of EVs isolation. (**B**) Relative protein concentration of EVs produced from naïve or primed cASCs (**C**) Representative electron micrograph of EVs isolation from naïve or primed cASCs. Bar = 100 nm. (**D**) Nanoparticle tracking analysis of EVs obtained from naïve or primed cASCs. The size of EVs ranged from 30–120 nm. (**E**) Representative image of western blot for the presence of CD63, CD9 and β-actin in cASCs and EVs isolation from naïve or primed cASCs. (**F**) The TSG-6, HGF and TGF-β levels in EVs from primed cASCs were higher than in EVs from naïve cASCs using western blot analysis. (**G**) In ELISA, higher levels of PGE2 concentration was measured in EVs of TNF-α and IFN-γ-primed cASC than in EVs of naïve cASC. Data are shown as mean ± S.D. (*ns* = Not Statistically Significant. *P < 0.05, **P < 0.01, ***P < 0.001, ****P < 0.0001 by unpaired two-tailed Student’s *t*-test).

EVs were isolated from the media of naive and primed cASCs. Interestingly, there was no difference in the relative EVs production of naive or primed cASCs (Fig. 2B) Specifically, EVs production occurred at a rate of 50 μg/ 5 × 10^5^ cASCs/72 h (data not shown) and TEM demonstrated that both the naïve and primed cASC-derived EVs were predominantly round and ranged in size from 30–120 nm (Fig. 2C,D). Both naive and primed cASC-derived EVs were also found to express exosomal markers such as CD63 and CD9, while the concentration of the cytosolic marker, β-actin, was low, but high in stem cells (Fig. 2E). Further, the primed EVs showed significantly higher expression of TSG-6, TGF-β, HGF, and PGE2 proteins than did naïve cASC-derived EVs (Fig. 2F).

### Effects of primed EVs enhanced Tregs *in vitro* and *in vivo*

**Figure 3:**
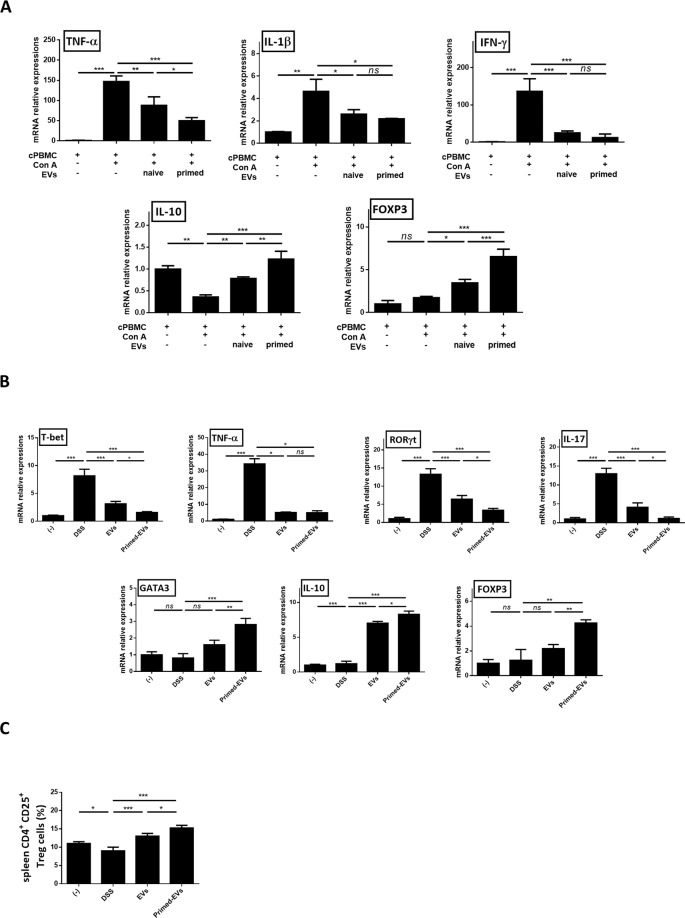
EVs from primed cASCs induce the expression of regulatory T cell *in vitro* and *in vivo*. (**A**) Con A-stimulated canine PBMCs were co cultured with EVs from naïve or primed cASCs for 48 h. TNF-α, IL-1β and IFN-γ mRNA levels were decreased and IL-10 and FOXP3 mRNA levels were increased in Con A-stimulated canine PBMC cultured with EVs. Furthermore, this effect was greater when the cells were treated with primed EVs. cPBMC+ : exist, Con A−: non-treated, Con A+ : treated, EVs−: absence (**B**) Changed in mRNA expression of Th1, Th2, Th17 and Tregs-related inflammatory mediators and (**C**) CD4^+^CD25^+^ Treg cell levels in spleen of DSS induced colitis mice. Results were shown as mean ± standard deviation. (*ns* = Not Statistically Significant *P < 0.05, **P < 0.01, ***P < 0.001 by one-way ANOVA analysis).

The expression of *TNF-α, IL-1β*, and *IFN-γ* decreased when Con A-stimulated cPBMCs were exposed to EVs and the expression of *IL-10* was increased. Furthermore, this effect was greater when the cells were treated with primed than with naive EVs (Fig. 3A). Tregs are known to play important roles in the alleviation of inflammation, and *FOXP3* is specifically expressed in naturally occurring Tregs. As such, the expression of *FOXP3* was increased in Con A stimulated-cPBMCs cultured with EVs, compared to the expression in cells cultured without EVs, and this effect was greater in activated cPBMCs cultured with primed EVs than in those cultured with naïve EVs (Fig. 3A).

In addition, Tregs are known to regulate inflammation and to alter the composition of lymphocytes, and thus, the level of Th1/Th17 and Th2/Treg cells was assessed using spleen tissue to confirm the overall lymphocyte regulation in IBD mice. The differentiation of Th1 lymphocytes is known to be associated with a specific transcription factor, T-bet; thus, the upregulation of Th1 activity in DSS-induced colitis and the reduction of Th1 activity after EVs treatment was confirmed by the analysis of *T-bet* expression. The reduction was more significant when primed EVs were administered relative to naïve. Similarly, the expression of *TNF-α* showed the same trend in DSS-induced colitis mice (Fig. 3B), although there was no significant difference in *TNF-a* between the primed and naïve EVs groups.

Next, the role of primed EVs in promoting the Th2 subset was investigated by analyzing IL-10, as well as the Th2 lineage transcription factor, GATA3. Accordingly, there was no significant change in the expression of *GATA3* or *IL-10* in the DSS-induced colitis group. However, levels of *GATA3* and *IL-10* were increased in the EVs-treated groups, an effect that was more significant in the primed than in the naïve EVs (Fig. 3B).

Moreover, the expression of retinoic acid-related orphan receptor γt (*RORγt*) revealed that EVs inhibited the differentiation of pathogenic Th17 effector cells, as RORγt regulates the development of Th17 cells. Further, primed EVs were found to suppress Th17 differentiation compared with naïve EVs. Expression of IL-17 was also significantly increased in the DSS-induced colitis but was downregulated in the primed and naïve EVs groups. It was also confirmed that primed EVs decreased IL-17 compared with naïve EVs (Fig. 3B). Likewise, the expression of *FOXP3* was increased in the EVs group relative to that in the PBS group. We also confirmed that primed EVs significantly increased *FOXP3* expression in the spleen relative to naïve EVs. Notably, there were significantly elevated CD4^+^CD25^+^ Tregs in primed EVs treated mice compared to that in naïve EVs-treated mice (Fig. 3C, Supplementary Fig. 3A).

### Induction of M2 macrophage polarization by primed EVs *in vitro* and *in vivo*

**Figure 4:**
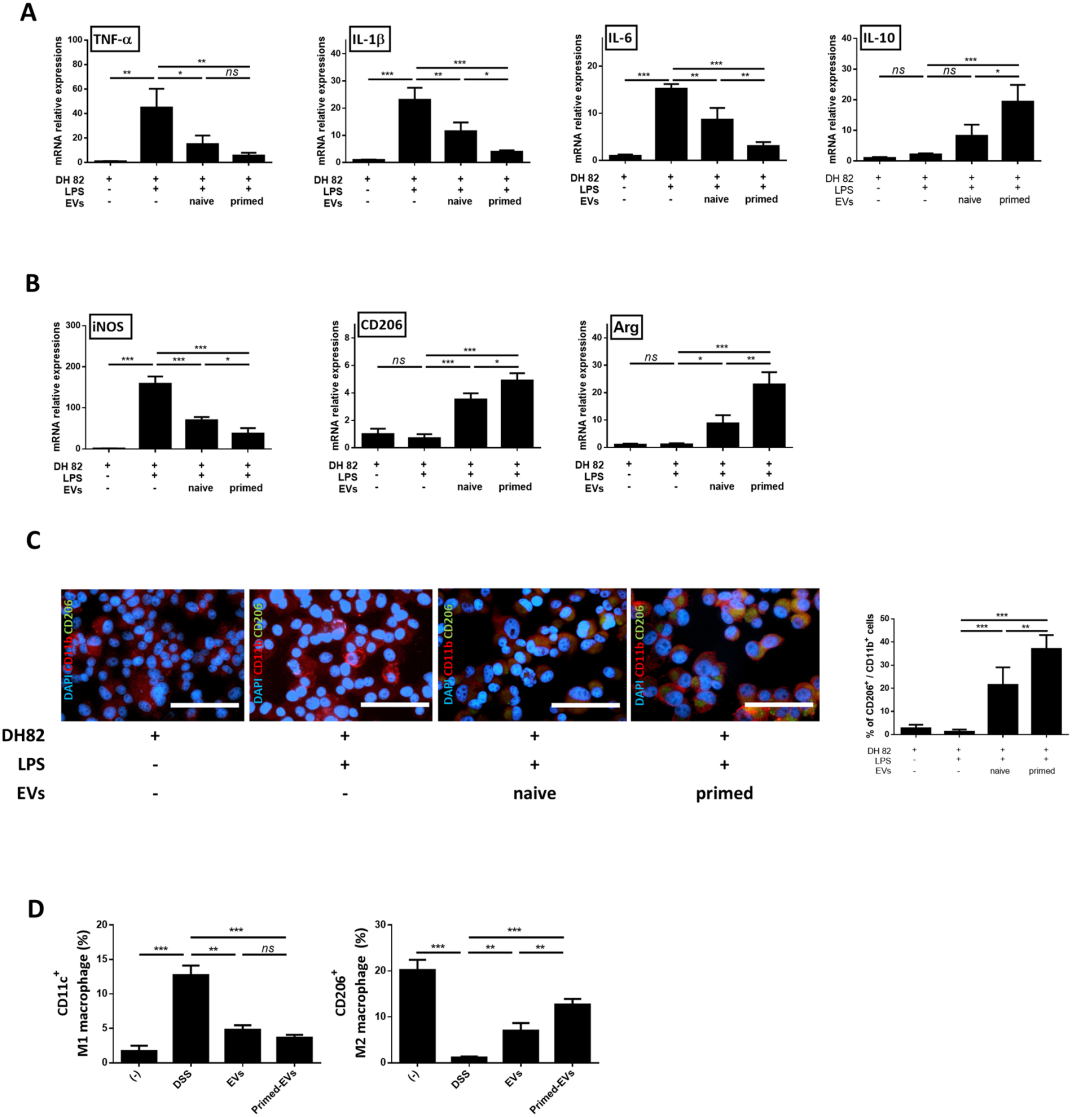
EVs from primed cASCs induce the expression of M2 macrophage marker *in vitro* and *in vivo*. LPS-stimulated DH82 were co-cultured with EVs from naïve or primed cASCs for 48 h. (**A**) Relative mRNA expression levels of TNF-α, IL-1β, IL-6 and IL-10 in RAW 264.7 and DH82 cells. (**B**) Relative mRNA expression of iNOS, CD206 and Arg are shown. DH82+ : exist, LPS−: non-treated, LPS+ : treated, EVs−: absence. (**C**) Representative immunofluorescence staining using anti-CD11b-PE or anti-CD206-FITC positive cell, and the calculated percentage of CD206-FITC positive cells among the CD11b-PE positive cell are shown. (**D**) CD11c^+^M1 and CD206^+^M2 peritoneal macrophage in DSS induced colitis mice model. Data are shown as mean ± S.D. (*ns* = Not Statistically Significant *P < 0.05, **P < 0.01, ***P < 0.001 by one-way ANOVA analysis).

The expression levels of *TNF-α*, *IL*-*1β*, *IL-6*, and *IL-10* were measured in LPS-stimulated DH82 cells to assess the immunomodulatory capacity of cASC-derived EVs. Expression of *TNF-α*, *IL-1β*, and *IL-6* was significantly reduced, while *IL-10* levels were significantly increased in the LPS-stimulated DH82 cultured with EVs relative to control. Furthermore, the effect was more significant when primed EVs were administered than when naïve cASC-derived EVs were administered (Fig. 4A). Next, to assess the ability of EVs to induce anti-inflammatory macrophage phenotypes, the expression of anti-inflammatory genes was examined in LPS-stimulated DH82 via RT-qPCR. Targets known to promote the differentiation of the M1 (inducible nitric oxide synthase [*iNOS*]) and M2 (*CD206* and *Arg*) phenotypes were selected. Accordingly, *iNOS* levels were significantly reduced in LPS-stimulated DH82 cells cultured with EVs relative to control. Furthermore, *iNOS* levels were significantly reduced in macrophages cultured with primed compared to naive EVs. Conversely, *CD206* and *Arg* levels were both significantly increased when the macrophages were cultured with EVs, and this effect was greater when the EVs were primed (Fig. 4B). Quantitative immunofluorescence examination of macrophage marker proteins also showed that the percentage of CD206^+^ M2 macrophages was significantly increased in LPS-stimulated DH82 cells cultured with EVs. Similarly, the primed EVs group exhibited a significantly higher percentage of CD206^+^ M2 macrophages than the naïve-EVs group. These results suggest that the EVs derived from the primed cASCs induced the M2 macrophage phenotype better than the naïve EVs. Thus, stimulating stem cells with inflammatory cytokines produces EVs with improved immunomodulatory properties (Fig. 4C). Furthermore, RAW 264.7 cells pretreated with LPS and co-cultured with primed EVs showed similar results to those of DH82 cells (Supplementary Fig. 2).

We next investigated the effects of primed EVs on the immune cell profile of DSS-induced colitis mice. In the peritoneal cavity, the levels of CD11^+^ M1 macrophages were significantly decreased in the EVs-treated mice relative to those in untreated IBD mice, but there was no difference between the mice treated with naïve and primed EVs. However, CD206^+^ M2 macrophages were decreased in DSS-treated mice but significantly increased in EVs-treated mice. Moreover, M2 macrophages were significantly more prevalent in mice treated with primed EVs than in naïve EVs-treated mice (Fig. 4D, Supplementary Fig. 3B).

### Primed cASC-derived EVs enhance regulatory T cells and regulate the M1/M2 balance in the inflamed colon

**Figure 5:**
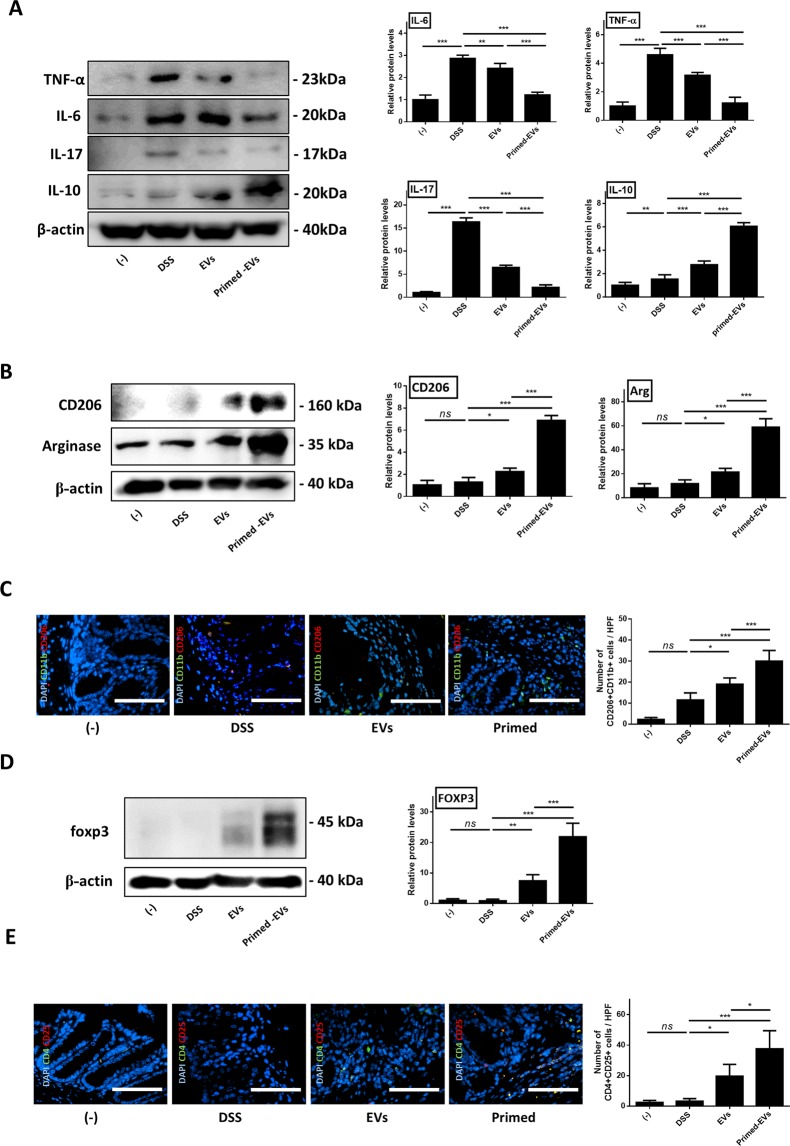
Changed in protein level of M2 macrophage and T reg-related mediator following treatment with primed EVs. (**A**) The primed EVs effectively inhibits inflammatory response in the inflamed colon. Relative expression levels of pro-inflammatory cytokines (TNF-α, IFN-γ and IL-17), anti-inflammatory cytokines (IL-10) were determined by western blot analysis. (**B**) Relative protein level of Arg, CD206 and beta-actin in colon tissue. (**C**) number of CD11b^+^CD206^+^ M2 cells in colon. (**D**) Relative protein level of FOXP3 in colon tissue. (**E**) Number of CD4^+^CD25^+^ Treg cells in colon were determined by FACS (*P < 0.05, **P < 0.01, ***P < 0.001 by one-way ANOVA analysis).

Next, the efficacy of EVs to attenuate DSS-induced colitis by regulating the immune response was assessed. Accordingly, RT-qPCR and western blot analysis demonstrated an increase in the expression of proinflammatory cytokines, such as TNF-α, IL-6, and IL-17, in the colon tissues of IBD mice and this was decreased in EVs-treated mice relative to that in untreated IBD mice. In particular, the reduction of IL-17 was most notable following administration of primed EVs. Further, the expression of anti-inflammatory cytokines, such as IL-10, was increased in the tissues of EVs-treated IBD mice compared to that in control mice; again, this was most significant in mice that received primed EVs (Fig. 5A, Supplement Fig. 4).

Inflamed colon tissue has a remarkable capacity for regeneration through a complex injury/repair process that includes inflammation. The observation that different macrophage subsets are associated with different stages of colon regeneration led us to investigate whether EVs treatment could influence macrophage polarization *in vivo*. This observation was accompanied by a significant increase in the expression of the M2 marker arginase 1 (*Arg1*) in the colon of mice treated with primed and naïve EVs. This effect was paralleled by the decreased expression of the M1 marker nitric oxide synthase 2 (*NOS2*). Furthermore, in mice that received primed EVs, the expression of M2 marker increased compared to that in those treated with naïve EVs (Fig. 5A,B, Supplement Fig. 4). The latter results were also confirmed by immunofluorescence, wherein the number of M2 macrophages (CD11b^+^CD206^+^ cells) was increased in the colon of IBD mice that received EVs, and this effect was more notable in the primed than in naïve EVs groups (Fig. 5C). Finally, western blotting and real-time PCR results showed that the expression of FOXP3 in the colon was also increased in EVs groups compared to that in the control group, and this effect was more significant in the primed EVs than in the naïve EVs groups (Fig. 5A,D, Supplement Fig. 4). The upregulation of Treg activities in DSS-induced colitis was further confirmed by analysis of CD4 and CD25 expression using immunofluorescence, and CD4^+^CD25^+^ cells were found to increase in the EVs groups, and the primed EVs group had more regulatory cells in the colon than the naïve EVs group (Fig. 5E).

## Discussion

Recently, studies have focused on the utility of stem cell-derived EVs as treatments for immune-mediated diseases^15,20^. EVs have been reported to mediate paracrine effects on receptor cells through membrane receptors or through intracellular incorporation or membrane fusion^21^. After fusion, various factors transferred by EVs can be translated in to protein, and these proteins affect cellular processes^22^. The cargo and function of EV depends on their producing cells, and it has been shown that also stress of cells affects EV content^23^.

TNF-α and IFN-γ are not only important inflammatory cytokines but are also mediators of the development of DSS-induced colitis^24–26^. Although TNF-α and IFN-γ are known to enhance the immunosuppressive properties of ASCs^12^, the therapeutic efficacy of EVs produced from ASCs pretreated with TNF-α and IFN-γ in colitis is unclear.

The purpose of this study was to evaluate the efficacy and immunomodulatory capacity of EVs derived from inflammatory cytokines primed canine stem cells in a colitis model with a normal immune system. Therefore, efficacy of EVs in the normal immune system was confirmed by inducing colitis in immunocompetent mice. The DSS-murine colitis model is very useful for evaluating novel therapeutics designed to promote epithelial repair and proliferation as well as mucosal wound repair in the presence of an acute inflammatory response^27^. In addition, the main advantage of this model is the ability to investigate the first immunological events associated with the induction of intestinal inflammation^28^.

In addition, we focused on the immune cells (such as macrophages and T lymphocytes) of mice after treatment with canine ASC-EVs. The canine ASCs were also immunoprivileged, partly due to the low expression of major histocompatibility complex class (MHC) II molecules^29^. And comprehensive proteomic analysis of EVs has not detected MHC II complex to date^30^. Similar strategies involving the administration of Heterogenous EVs to immunocompetent animal models have been adopted by several groups, and no obvious cross-species-induced immunological responses have been reported^31^. Therefore, we evaluated the efficacy of canine stem cell-derived EVs in a mouse model before application to canine patients.

According to the results of injecting EVs into mice, biodistribution occurred mainly in the lungs and liver when injecting intravenously (IV), whereas infusion by intraperitoneally(IP) was evenly distributed in liver, pancreas and gastrointestinal tract^32,33^. Therefore, IP is considered a suitable method to confirm the therapeutic effect of EVs in the gastrointestinal disease^34–36^. However, little has been done about the distribution pattern of primed EVs in inflamed colon of murine model.

Some studies have shown that EVs generated from priming stem cells reach more damaged tissues, increasing the protection effect^37^. However, unlike other studies, this study demonstrated that similar amount of primed EVs and naïve EVs have been found in colon, and that primed EVs is very effective in mitigating inflammation. This suggested that primed EVs are more efficient at treating colitis. However, further research is needed on how primed EVs have increased their ability to mitigate colitis.

Under immunofluorescence microscope, in the group injected with EVs, a number of cells with EVs in the cytoplasm were identified in mucosa and submucosa. These infiltrated inflammatory cells, such as lymphocytes, neutrophils, monocytes and macrophages, which play an important role in regulating inflammatory responses in colon, are implicated in IBD etiology^38^. Among them, T regs and macrophages are able to control the functions of neutrophils and other inflammatory cells, which have been widely shown to be involved in process of tissue repair^39,40^. Therefore, it is very important to clarify the correlation between primed EVs and these immune cells.

The immunomodulatory capacity of stem cells is not entirely innate, and the immunosuppressive ability is known to be derived from inflammatory cytokines in the external environment^41^. Although studies have been made on primed stem cells^9,42^, the effects on pretreatment of stem cell-derived EVs are lacking, and further research is needed on therapeutic effects in *in vivo* immune mediated models. In this experiment, pre-stimulation of stem cells with TNF-α and IFN-γ significantly increased TSG-6, TGF-β, HGF and PGE2 expression in EVs. TSG-6 is known as a potent inhibitor of neutrophil migration, suppresses inflammatory signaling in tissue-resident immune cells, and polarizes macrophage to the M2 phenotype^43,44^. And Kota *et al*. reported that TSG-6 produced by human stem cells enhancing T reg regeneration in diabetes model^45^. Similarly, TGF-β, which is a major pluripotential cytokines with a pronounced immunosuppressive effect, inhibits macrophage activation by polarizing macrophage from M1 to M2^46,47^, and supports the maintenance of regulatory function, and homeostasis in peripheral CD4+CD25+ T reg cells^48^. HGF exerts anti-inflammatory activities via immune cell regulation, including differentiation and cytokine production, and T cell effector function^41,49^. And Choi *et al*. reported that HGF has a healing effect on damaged tissues by polarizing Macrophage from M1 to M2^50^. In addition, Benkhoucha *et al*. showed that HGF is a potent immunomodulatory factor that inhibits dendritic cell function along with differentiation of IL-10–producing Tregs^51^. PGE2 alters the cytokine secretion profile of the T cell subset to change from a proinflammatory to an anti-inflammatory environment^52,53^. We confirmed the efficacy of ASC in murine-derived macrophage cell lines in inflammatory environments and demonstrated that PGE2 secreted from stem cells is a key factor in polarizing macrophage^12,54^. While further study is needed to assess whether other miRNAs, mRNAs, or proteins also mediate the immunomodulatory effect of the primed cASC-derived EVs, our findings demonstrate that proinflammatory cytokine stimulation was sufficient to induce the cASCs to release EVs containing more potent immunoregulatory factors. Furthermore, our results showed that EVs could both shape the host response to effectively polarize macrophage from M1 to M2 and switch T cells from a Th1/Th17 immune profile towards a Th2/Treg immune profile. In addition, primed EVs were found to increase both T reg and M2 in inflamed colon.

Taken together, primed EVs were thought to increase the ability to alleviate colitis by enhancing the regulation of immune cells. However, more research is needed to determine which of these two immune cells regulates more preferentially.

Previous other studies showed that stem cell derived EVs could modulate monocyte toward M2-like phenotype and macrophage colony-stimulating factor polarized M2 monocytes could induce T regs^55,56^. However, in other study, expanded T regs have the capacity to induce phenotypical and functional changes in monocytes that might be crucial for tolerance induction in transplantation and the prevention/treatment of autoimmune diseases^57^. Our previous study found that in the DSS-induced mouse colitis model, it is important to convert the polarization of macrophage to M2 type in controlling inflammation^11^. However, other studies have demonstrated that increasing T regs in the colon is a major factor in relieving inflammation^58^. Although which is more dominant is still controversial, monocytes and T regs may interact with each other, and it is obvious that T regs and M2 play a major regulator in colitis alleviation.

This study is important in that it provides an essential basis for the application of canine stem cells to canine IBD patients. Spontaneous lymphoprotein-plasmocytic colitis in dogs and human IBD share several histopathological and molecular features^59^. Thus, spontaneous canine IBD is an appropriate model for the study of the multifactorial pathogenesis of IBD, EVs immune modulation mechanisms, determining dose equivalence, and the biological effect of stem cell-derived EVs therapies in refractory IBD^60^. Therefore, this study is important not only in terms of increasing the immune modulatory effect of stem cell-derived EVs but also as a basis for future translational research. Thus, the present study provides a basis for further research into the potential use of EVs to treat immune-mediated diseases in canines. Immune-related diseases have serious consequences not only in canines but also in humans. This research will be of great interest to researchers in various scientific fields including canine and human basic and medical research.

As far as we know, this is the first time to confirm the therapeutic effect by applying EVs derived from stem cells pretreated with inflammatory cytokines such as TNF-α and IFN-γ to colitis murine model. In addition, the present study demonstrated that TNF-α/IFN-γ pretreatment enhanced the ability of cASC-derived EVs to induce M2-macrophage polarization, enhance T regs, and to regulate the production of both pro- and anti-inflammatory cytokines in inflamed colon. Our findings strongly suggest that an inflammatory stimulus may be fundamental for inducing the release of immunomodulatory EVs from cASCs and support further investigation of primed cASC-derived EVs as potential therapeutic agents to treat immune-related diseases as well as IBD. This experiment is an important data to assess the effects of stem cell-derived EVs before they are applied to not only dog but also human patients.

## Materials and Methods

All Animal experimental procedures were approved by the Institutional Animal Care and Use Committee of (SNU protocol no. SNU-190117-4), Republic of Korea, and all protocols were in accordance with approved guidelines

### Canine adipose tissue derived mesenchymal stem cells (cASC) activation with TNF-α and IFN-γ

cASCs were used and isolated as previously described^12^. The cASCs were seeded in 6-well plates (5 × 10^5^ cells/well) and cultured in Dulbecco’s modified Eagle’s medium with 4.5 g/L glucose (DMEM; PAN-Biotech, Aidenbach, Germany) containing 10% Exo-free fetal bovine serum (FBS; PAN-Biotech) and 1% penicillin-streptomycin (PS; PAN-Biotech). After 6 h, the cells were stimulated for 24 h with TNF-α (20 ng/mL; PROSPEC, Ness Ziona, Israel) and IFN-γ (20 ng/mL; Kingfisher Biotech, Saint Paul, MN). The morphology and viability of cASCs were assessed prior to their use in the experiments (CCK-8; Donginbio, Seoul, South Korea). In addition, the expression of several stem cell markers on these cells was determined by flow cytometry (fluorescence‐activated cell sorter (FACS) Canto II; BD Pharmingen, San Diego, CA, USA) using the CD90-PE (Invitrogen, Carlsbad, CA), CD44-FITC (Invitrogen, Carlsbad, CA), CD29-FITC (BD Biosciences, Franklin Lakes, NJ), CD73-PE (BD Biosciences, Franklin Lakes, NJ), CD45-FITC (BD Biosciences, Franklin Lakes, NJ), and CD34-PE (BD Biosciences, Franklin Lakes, NJ) antibodies. Their cell-differentiation capacity was assessed using the StemPro Osteogenesis, Adipogenesis, and Chondrogenesis Differentiation Kits (Thermo Fisher Scientific, Waltham, MA) according to the manufacturer’s instructions. Adipocytes, osteocytes, and chondrocytes were identified via Oil-Red-O, Alizarin-red, and Alcian-blue staining (Sigma-Aldrich, St. Louis, MO) according to the manufacturer’s instructions. Additionally, cells were harvested for RNA extraction.

### cASC-derived EVs isolation and characterization

Naive and primed cASCs (5 × 10^5^ cells/well) were maintained in 2 mL of DMEM containing 10% Exo-free fetal bovine serum and 1% PS for 72 h. The medium was then harvested on ice, centrifuged (300 *g*, 4 °C, 10 min), transferred to fresh tubes, and centrifuged once more (2000 *g*, 4 °C, 30 min), after which the supernatant was filtered through a 0.22-μm filter (Millipore). The resultant supernatants were transferred to fresh tubes and centrifuged twice at 110,000 *g* (4 °C, 80 min) in an Avanti Centrifuge J-26XP equipped with a 70Ti rotor (Beckman Coulter, Brea, CA), with a PBS washing step between the two centrifugation steps. The final pellet was resuspended in 100 μL PBS and sterilized via filtration through a 0.22-μm filter (Fig. 2A). The total protein concentration of each EVs preparation was quantified via a BCA assay, and the preparations were stored at −80 °C until further use. The morphology of the purified EVs was characterized via transmission electron microscopy (TEM). Briefly, a 10 μL EVs suspension was placed on a clean piece of parafilm. A 300-mesh Formvar-carbon-coated electron microscopy grid was then floated on the drop with the coated side facing the suspension and left to absorb for 20 min at room temperature. The grid was transferred to a 100-μL distilled water and incubated for 2 min, before being transferred to 50 μL 2% uranyl acetate and left to incubate for 10 min for negative staining. The grid was then observed using a LIBRA 120 (Carl Zeiss, Germany) at 120 kV. The particle size distribution was measured using a Zeta-Potential & Particle Size Analyzer (ELSZ-1000 ZS, Otuka Electrons, Japan). In addition, the expression of several EVs markers on these EVs was determined by western blot analysis.

### Western blot analysis

Total proteins from EVs, cASCs, and colon tissues were extracted using the PRO-PREP Protein Extraction Kit (iNtRON Biotechnology, Seongnam, South Korea) and measured using the Bio-Rad DC Protein Assay Kit (Bio-Rad Laboratories, Hercules, CA, USA). The total protein content in each 20 μg sample was subjected to SDS-PAGE and immunoblotting with antibodies against TSG-6 (Santa Cruz Biotechnology, CA, USA), COX-2(Santa Cruz Biotechnology), HGF, beta-actin(Santa Cruz Biotechnology), CD206 (Santa Cruz Biotechnology), Arginase (Santa Cruz Biotechnology), FOXP3 (Santa Cruz Biotechnology), TGF-β (Cusabio Biotech, Wuhan, China), CD63 (LSBio, Seattle, WA, USA), CD9 (GeneTex, Irvine, CA, USA), beta-actin (Santa Cruz Biotechnology), TNF-α (Cusabio Biotech), IL-6 (Cusabio Biotech), IL-10 (ABclonal Tech, MA, USA) and IL-17 (ABclonal Tech).

### ELISA

The PGE2 content of the cASC-secreted EVs was detected using a PGE2 ELISA Kit (Cusabio Biotech) according to the manufacturer’s instructions.

### Co-culture experiments

RAW 264.7 cells, a murine macrophage-like cell line, and DH82 cells, a canine macrophage-like cell line, were purchased from the Korean Cell Line Bank (Seoul, Korea). RAW 264.7 and DH82 cells were seeded in 6-well plates (1 × 10^6^ cells/well) in triplicate and incubated for 24 h. After adherence to the plates was confirmed, the RAW 264.7 and DH82 cells were treated with LPS (200 ng/mL; Sigma-Aldrich) or control for 24 h. Similarly, with the consent of the owners, 10 mL of blood was obtained from each of 3 dogs and canine peripheral blood mononuclear cells (cPBMCs) were obtained using ficoll-plaque PLUS (Sigma-Aldrich) according to the manufacturer’s instructions. cPBMCs were seeded in 6-well plates (1 × 10^6^ cells/well) in triplicate, incubated for 24 h, and exposed to Con A (5 μg/mL) or control for 24 h. Next, the medium was removed and replaced with media containing EVs (50 μg/well) derived from naive (unstimulated) or primed (stimulated) cASCs. Next, the cells were incubated for 48 h and then harvested for RNA extraction and immunofluorescence analysis.

### DSS-induced colitis mice

Male C57BL/6 J mice aged 6 weeks were purchased from Nara Biotech (Seoul, Korea) and housed under controlled temperature, humidity, and light cycle conditions. Acute colitis was induced by the administration of 3% dextran sulfate sodium (DSS; Molecular weight 36–59 kDa; MP Biomedicals, Santana, CA, USA) from day 0 to day 7 in the drinking water *ad libitum*. The water was no longer treated after day 8, as previously described^61^ (Fig. 1A). Mice were randomly divided into the following four groups (n = 4–6 mice/group): (1) Control (n = 4); (2) DSS(n = 6); (3) DSS with EVs (n = 6); (4) DSS with primed cASC-derived EVs (n = 6). At days 1, 3, and 5, mice were intraperitoneally injected with 100 µg EVs, from either naïve or primed cASCs, diluted in 200 µL PBS or vehicle control (PBS). Mice were euthanized at day 10. These procedures were approved by the Institutional Animal Care and Use Committee of Seoul National University (SNU), Republic of Korea, and all protocols were in accordance with approved guidelines (SNU; protocol no. SNU-190117-4).

### Evaluating colitis severity

The severity of colitis was assessed daily by clinical disease activity scoring, including assessing general activity, stool consistency, presence of fecal blood, and weight loss. The entire colon was removed from the cecum to the anus, and colon length was measured. Overall disease severity was assessed as follows: general activity: 0 (normal), 2 (mild depression), or 4 (severely depressed); stool consistency: 0 (normal), 1 (loose stool), or 2 (diarrhea); fecal blood: 0 (no blood), 1 (visual pellet bleeding), or 2 (gross bleeding, blood around anus); weight loss: 0 (no loss), 1 (1–5%), 2 (5–10%), 3 (10–15%), or 4 (>15%).

### Histological analysis

The colon was fixed in 10% formalin, embedded in paraffin, and sectioned at 3 μm. To assess colonic damage microscopically, the slides were stained with H&E. Histological scores are provided in Table 1. The degree of inflammatory cell infiltration and the degree of barrier integrity were combined to give a histological inflammatory score. Because DSS injury varies, two slides from each section of the colon were assessed per mouse, and at least three areas on each slide were examined.

**Table 1:** Histological inflammatory score.

	Infiltration	Epithelium
0	No infiltration	Normal morphology
1	Infiltrate around crypt basis	Loss of goblet cells
2	Infiltrate reaching to lamina muscularis mucosa layer	Loss of goblet cell in large areas
3	Extensive infiltration reaching the muscularis mucosa with abundant edema	Loss of crypts
4	Infiltration of the submucosa layer	Loss of crypts in large areas

### Immunofluorescence analysis

RAW 264.7 and DH82 were washed three times with DPBS and fixed with 4% paraformaldehyde for 20 min at room temperature. After washing with PBS, the cells were permeabilized for 1 h with 0.2% Triton X-100 (Sigma-Aldrich), then blocked for 1 h at room temperature with 2% FBS. The cells were incubated sequentially with PE-conjugated CD11b (1:200) and FITC-conjugated CD206 (1:200; Santa Cruz Biotechnology) antibodies at 4 °C overnight in the dark. Finally, the cells were washed three times with PBS and mounted. Paraffin sections were cut at a thickness of 4 μm for immunostaining. Sections were deparaffinized and rehydrated, and antigen retrieval was carried out in 10 mM citrate buffer. Sections were then washed and blocked with blocking buffer containing 5% bovine serum albumin and 0.3% Triton X-100 for 1 h. The sections were then incubated overnight at 4°C with mouse monoclonal FITC-conjugated CD4 (1:100; Santa Cruz Biotechnology) and PE-conjugated CD25 (1:100; Santa Cruz Biotechnology) or FITC-conjugated CD11b (1:100; Abcam) and PE-conjugated CD206 (1:100; Santa Cruz Biotechnology). The colon sections were washed three times. All samples were mounted using Vectashield mounting medium containing 4′,6-diamidino-2-phenylindole (DAPI; Vector Laboratories, Burlingame, CA, USA). The samples were observed using an EVOS FL microscope (Life Technologies, Darmstadt, Germany). Immunoreactive cells were counted in 20 random fields per group, and the percentage of CD206^+^CD11b^+^ positive cells and CD25^+^CD4^+^ positive cells was calculated in colon sections from the same mice.

### RNA extraction, cDNA synthesis, and RT-qPCR

RNA was extracted from RAW 264.7, DH82, cPBMCs, cASCs, mouse spleen tissue, and colon tissue using the Easy-BLUE Total RNA Extraction Kit (iNtRON Biotechnology). cDNA was then synthesized from the isolated RNA using the LaboPass M-MuLV Reverse Transcriptase Kit (Cosmogenetech, Seoul, South Korea) according to the manufacturer’s instructions. The resultant cDNA samples were then subjected (in duplicate) to qRT-PCR using AMPIGENE qPCR Green Mix Hi-ROX with SYBR Green Dye (Enzo Life Sciences, USA) according to the manufacturer’s instructions. The mRNA expression levels were normalized to that of glyceraldehyde 3-phosphate dehydrogenase (*GAPDH*). The sequences of primers used throughout this study are given in Table 2.

**Table 2:** Sequences of PCR primers used in this study.

Gene	Forward (5′-3′)	Reverse (5′-3′)	Reference
mGAPDH	AGTATGTCGTGGAGTCTACTGGTGT	AGTGAGTTGTCATATTTCTCGTGGT	^62^
mTNF-α	CCAGGAGAAAGTCAGCCTCCT	TCATACCAGGGCTTGAGCTCA	^63^
mIFN-γ	GATGCATTCATGAGTATTGCCAAGT	GTGGACCACTCGGATGAGCTC	^64^
mIL-1β	CACCTCTCAAGCAGAGCACAG	GGGTTCCATGGTGAAGTCAA	^65^
mIL-6	TCCAGTTGCCTTCTTGGGAC	GTACTCCAGAAGACCAGAGG	^66^
mIL-10	TGGCCCAGAAATCAAGGAGC	CAGCAGACTCAATACACACT	^54^
miNOS	AAAGGAAATAGAAACAACAGGAACC	GCATAAAGTATGTGTCTGCAGATGT	^67^
mCD206	AACGGAATGATTGTGTAGTTCTAGC	TACAGGATCAATAATTTTTGGCATT	^43^
mArg1	CAGAAGAATGGAAGAGTCAG	CAGATATGCAGGGAGTCACC	^68^
mFOXP3	TTGGCCAGCGCCATCTT	TGCCTCCTCCAGAGAGAAGTG	^69^
mIL-17	GAGAGCTGCCCCTTCACTTTCA	GGCTGCCTGGCGGACAAT	^70^
mGATA3	AGGTGCATGACGCGCTGGAG	GGAGTGGCTGAAGGGAGAG	^70^
mT-bet	ACCTCTTCTATCCAACCAGTATCC	GAGGTGTCCCCAGCCAGTA	^70^
mPORγt	GAAGGCAAATACGGTGGTGTGG	GCTGAGGAAGTGGGAAAAGTC	^70^
cGAPDH	TTAACTCTGGCAAAGTGGATATTGT	GAATCATACTGGAACATGTACACCA	^12^
ciNOS	GAGATCAATGTCGCTGTACTCC	TGATGGTCACATTTTGCTTCTG	^71^
cCD206	GGAAATATGTAAACAGGAATGATGC	TCCATCCAAATAAACTTTTTATCCA	^71^
cArg	AAATTATGTCCTGTCCCCTTTCTAC	TTTAAGTTGAATCTTTTTCCTGTGG	^12^
cTNF-α	TCATCTTCTCGAACCCCAAG	ACCCATCTGACGGCACTATC	^72^
cIL-1β	AGTTGCAAGTCTCCCACCAG	TATCCGCATCTGTTTTGCAG	^72^
cIL-6	ATGATCCACTTCAAATAGTCTACC	AGATGTAGGTTATTTTCTGCCAGTG	^12^
cIL-10	ATTTCTGCCCTGTGAGAATAAGAG	TGTAGTTGATGAAGATGTCAAGCTA	^73^
cFOXP3	AAACAGCACATTCCCAGAGTTC	AGGATGGCCCAGCGGATCAG	^61^

### Statistical analysis

All experiments were performed in triplicate for each condition and are expressed as the mean ± standard deviation, representative of three independent experiments with similar results. Statistical comparisons between two groups were performed using an unpaired two-tailed Student’s *t*-test. Differences between multiple groups were statistically analyzed using a one-way analysis of variance (ANOVA) and a Tukey’s multiple comparisons test. P values < 0.5 were considered to indicate statistical significance. All statistical analyses were performed using GraphPad Prism v6.01 (GraphPad Software Inc., La Jolla, CA, USA).

## Supplementary information

Supplementary information.

## Data Availability

All data generated or analysed during this study are included in this published article (and its Supplementary Information files).
