# Author Correction: TNF-α and INF-γ primed canine stem cell-derived extracellular vesicles alleviate experimental murine colitis

**DOI:** 10.1038/s41598-020-63115-3

**Published:** 2020-04-30

**Authors:** Ju-Hyun An, Qiang Li, Dong-Ha Bhang, Woo-Jin Song, Hwa-Young Youn

**Affiliations:** 1 Labolatory of Veterinary Internal Medicine, Department of Veterinary Clinical Science, College of Veterinary Medicine and Research institute for Veterinary Science, Seoul National University, 1 Gwanak-ro, Gwanak-gu, Seoul 08826 Republic of Korea; 2 Department of Molecular Cell Biology, , Samsung Biomedical Research Institute, Sungkyunkwan University School of Medicine, Suwon-si, Gyeonggi-do 16419 Republic of Korea

Correction to: *Scientific Reports* 10.1038/s41598-020-58909-4, published online 07 February 2020

In this Article, the legend of Figure 1 is incorrect:

“cASC-derived EVs ameliorates DSS-induced colitis in mice. Naïve or primed EVs (100 μg in 100 μl PBS) or vehicle control (100 μl PBS) were intraperitoneally (IP) injected into intraperitoneally on days 1, 3 and 5 after mice fed with 3% DSS.”

should read:

“cASC-derived EVs ameliorates DSS-induced colitis in mice. Naïve or primed EVs (100 μg in 200 μl PBS) or vehicle control (200 μl PBS) were intraperitoneally (IP) injected into intraperitoneally on days 1, 3 and 5 after mice fed with 3% DSS.”

In addition, the legend of Figure 4 is incorrect:

“Relative mRNA expression levels of TNF-α, IL-1β, IL-6 and IL-10 in RAW 264.7 and DH82 cells”

should read:

“Relative mRNA expression levels of TNF-α, IL-1β, IL-6 and IL-10 in DH82 cells”

Also, the legend of Figure 5 is incorrect:

“The primed EVs effectively inhibits inflammatory response in the inflamed colon. Relative expression levels of pro-inflammatory cytokines (TNF-α, IFN-γ and IL-17), anti-inflammatory cytokines (IL-10) were determined by western blot analysis”

should read:

“The primed EVs effectively inhibits inflammatory response in the inflamed colon. Relative expression levels of pro-inflammatory cytokines (TNF-α, IL-6 and IL-17), anti-inflammatory cytokines (IL-10) were determined by western blot analysis”

Finally, there is a typographical error in the Materials and Methods section under subheading ‘Statistical Analysis’ where,

“Differences between multiple groups were statistically analyzed using a one-way analysis of variance (ANOVA) and a Tukey’s multiple comparisons test. P values < 0.5 were considered to indicate statistical significance.”

should read:

“Differences between multiple groups were statistically analyzed using a one-way analysis of variance (ANOVA) and a Tukey’s multiple comparisons test. P values < 0.05 were considered to indicate statistical significance.”

