# Supplementary material for: TNF-α and INF-γ primed canine stem cell-derived extracellular vesicles alleviate experimental murine colitis
Source: Sci Rep. 2020 Feb 7;10:2115. doi: 10.1038/s41598-020-58909-4 (PMC7005871; doi:10.1038/s41598-020-58909-4)
Supplement: Supplementary file 1 — Supplementary information. [file 41598_2020_58909_MOESM1_ESM.pdf]

TNF-α and INF-γ primed canine stem cell-derived extracellular vesicles alleviate experimental murine colitis

Ju-Hyun AN[1], Qiang LI[1], Dong-Ha BHANG[2], Woo-Jin Song[1*] and Hwa-Young YOUN[1*]

[1]Labolatory of Veterinary Internal Medicine, Department of Veterinary Clinical Science, College of Veterinary Medicine and Research institute for Veterinary Science, Seoul National University, 1 Gwanak-ro, Gwanak-gu, Seoul 08826, Republic of Korea

[2]Department of Molecular Cell Biology, Samsung Biomedical Research Institute, Sungkyunkwan University School of Medicine, Suwon-si, Gyeonggi-do 16419, Republic of Korea

[*]Author Email: Ju-Hyun AN: ajh3212@snu.ac.kr, Qiang LI: liqiang8589@snu.ac.kr, Dong-Ha BHANG: Bhangd77@gmail.com,

[*]Corresponding Author: Woo-Jin SONG (E-mail: woogin1988@snu.ac.kr) and Hwa-Young YOUN (Tel: +82-2-880-1266, E-mail: hyyoun@snu.ac.kr)

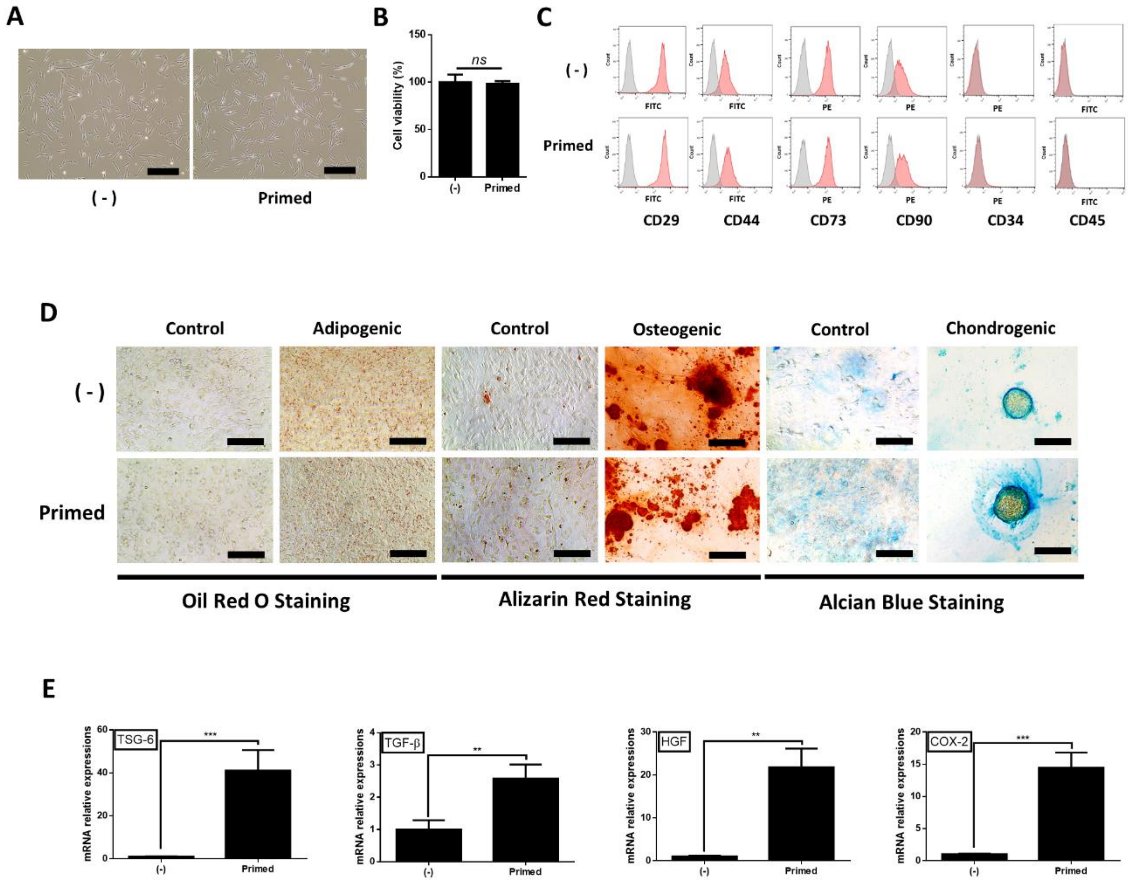

Supplementary Figure 1. Characterization and immunomodulatory factors of naïve- and primed cASCs (A) Representative phase contrast images of cASCs incubated with TNF-α and IFN-γ at concentration of 20 ng/mLfor 24 h. Scale bars = 200 μm. (B) Cell viability of cAT-MSCs incubated with TNF-α and IFN-γ at concentration of 20 ng/mLfor 24 h was determined by CCK-8 assay. (C) Naïve and primed cASCs have high expression of CD29, CD44, CD73, and CD90 and low expression of CD34 and CD45. (D) Naïve and primed cASCs have the ability to differentiate into adipocyte (Oil Red O staining), osteocytes (Alizarin Red S staining), and chondrocytes (Alcian Blue staining), Scale bars = 200 μm. Data are shown as mean ± S.D. (*ns* = Not Statistically Significant by unpaired two-tailed Student's *t*-test)

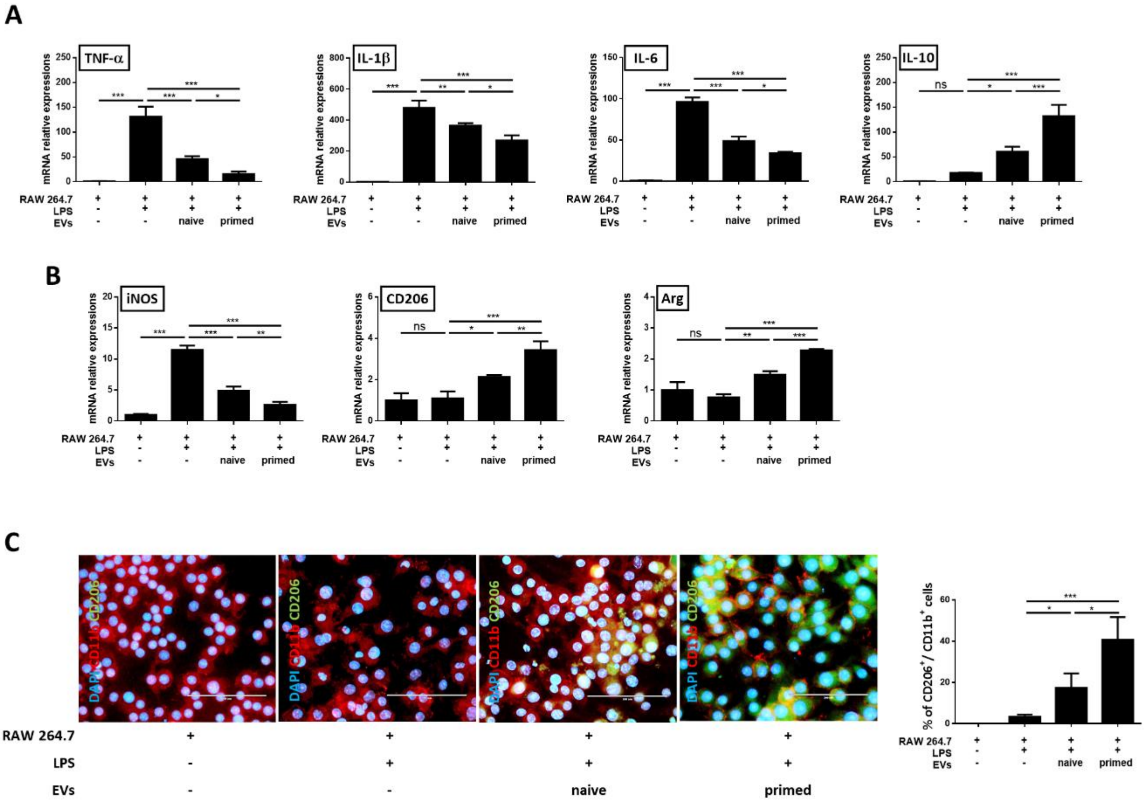

Supplementary figure 2. EVs from primed cASCs induce the expression of M2 macrophage marker in RAW 264.7 cells LPS-stimulated RAW 264.7 cells were co-cultured with EVs from naïve or primed cASCs for 48 h. (A) Relative mRNA expression levels of TNF-α, IL-1β, IL-6 and IL-10 in RAW 264.7 and RAW 264.7 cells. (B) Relative mRNA expression of iNOS, CD206 and Arg are shown. RAW 264.7 +: exist, LPS -: non-treated, LPS +: treated, Exosome -: absence. (C) Representative immunofluorescence staining using anti-CD11b-PE or anti-CD206-FITC positive cell, and the calculated percentage of CD206-FITC positive cells among the CD11b-PE positive cell are shown. Data are shown as mean ± S.D. Scale bars = 200 μm (*ns* = Not Statistically Significant $^*P < 0.05$, $^{**}P < 0.01$, $^{***}P < 0.001$ by one-way ANOVA analysis)

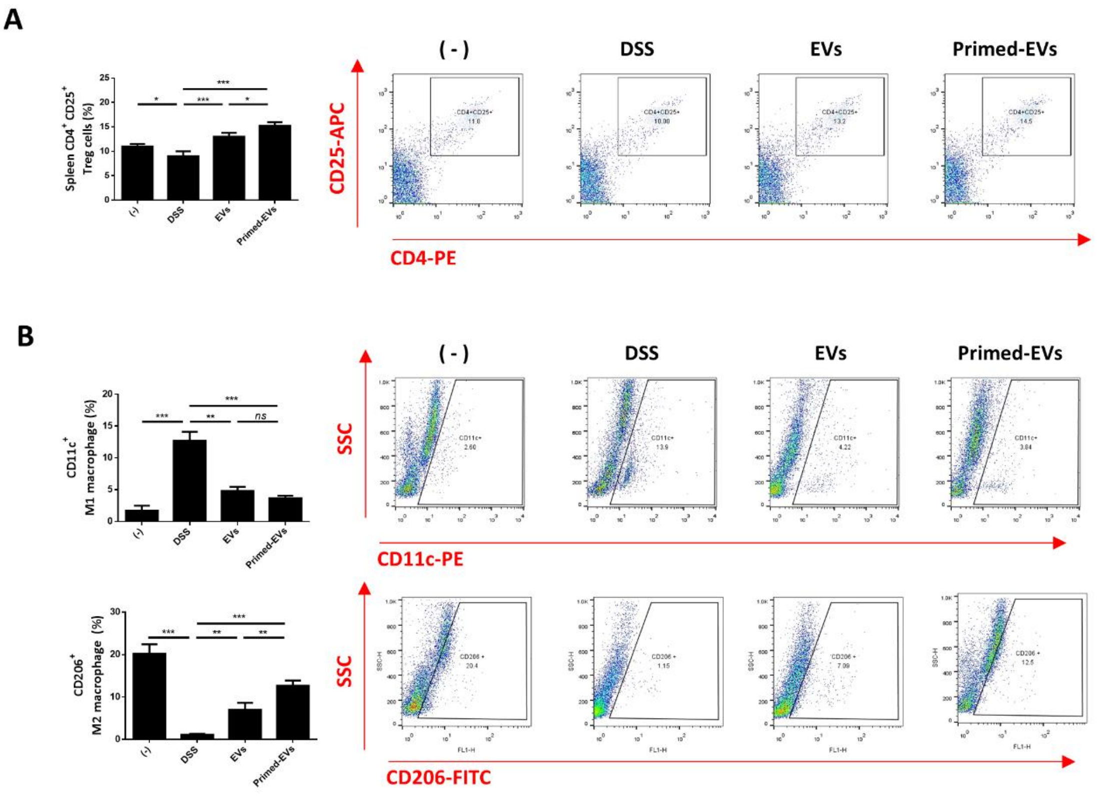

Supplementary figure 3. Primed cASCs derived EVs enhance regulatory T cells and regulate the M1/M2 balance in colitis mice model. Overall M2 macrophage and Tregs were significantly increased in primed EVs injected colitis mice model compared with naïve-EVs injected colitis mice model. (A) M1($CD11c^{+}$cells) and M2 macrophage ($CD206^{+}$ cells) level in DSS induced colitis mice peritoneal cavity (B) Tregs ($CD4^{+}CD25^{+}$ cells) levels in spleen of DSS induced colitis mice. FACS plots (right panel) show representative examples and bar graphs (left panel) represent mean values $\pm$ SD (*ns* = Not Statistically Significant *P < 0.05, **P < 0.01, ***P < 0.001 by one-way ANOVA analysis)

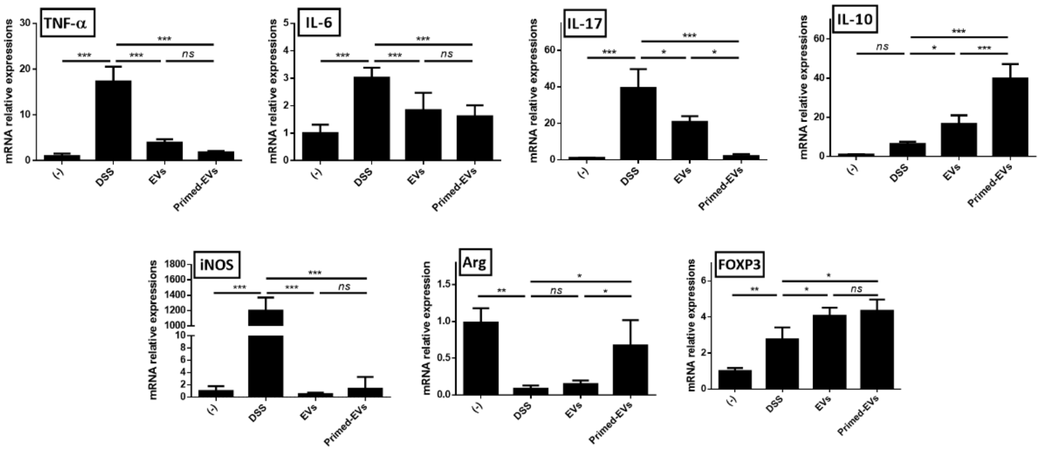

Supplementary figure 4. Changed in mRNA expression of M2 macrophage and T reg-related mediator following treatment with primed EVs. The primed EVs effectively inhibits inflammatory response in the inflamed colon. mRNA expression levels of pro-inflammatory cytokines (TNF-α, IFN-γ and IL-17), anti-inflammatory cytokines (IL-10), macrophage related markers (*iNOS, Arg*) and Tregs related marker (*FOXP3*) in colon tissue were determined by RT-qPCR. (*P < 0.05, **P < 0.01, ***P < 0.001 by one-way ANOVA analysis)
